# Native high-resolution 3D SSFP MR angiography for assessing the thoracic aorta

**DOI:** 10.1186/1532-429X-16-S1-P165

**Published:** 2014-01-16

**Authors:** Florian von Knobelsdorff, Henriette Gruettner, Ralf F Trauzeddel, Andreas Greiser, Jeanette Schulz-Menger

**Affiliations:** 1 Cardiac MRI, Charité Medical Faculty, Berlin, Germany; 2 Cardiology and Nephrology, HELIOS Clinics, Berlin, Germany; 3 Siemens Healthcare, Erlangen, Germany

## Background

To omit risks of contrast agent administration, native magnetic resonance angiography (MRA) is desired for assessing the thoracic aorta. Aim was to evaluate a native steady-state free-precession (SSFP) 3D MRA in comparison to contrast-enhanced MRA as the gold standard.

## Methods

Seventy-six prospective patients with known or suspicion of thoracic aortic disease underwent MRA at 1.5T using i) native 3D SSFP MRA with ECG and navigator gating and high isotropic spatial resolution (1.3 × 1.3 × 1.3 mm 3) and ii) conventional contrast-enhanced ECG-gated gradient echo 3D MRA (1.3 × 0.8 × 1.8 mm 3). Datasets were compared at 9 aortic levels regarding image quality (score 0-3: 0 = poor, 3 = excellent) and aortic diameters, as well as observer dependency and final diagnosis.

## Results

Native 3D-MRA was acquired successfully in 70/76 subjects (mean acquisition time 8.6 ± 2.7 min), while irregular breathing excluded 6/76 subjects. Aortic diameters agreed close between both methods at all aortic levels (r = 0.99; bias ± SD -0.12 ± 1.2 mm) with low intra- and inter-observer dependency (intraclass correlation coefficient 0.99). Native MRA studies resulted in the same final diagnosis as the contrast-enhanced MRA. Mean image quality score was superior with native compared to contrast-enhanced MRA (2.4 ± 0.6 vs. 1.6 ± 0.5; p < 0.001). The Figure provides a set of examples acquired with both techniques (left: native, right: contrast-enhanced).

## Conclusions

Accuracy of aortic size measurements, certainty in defining the diagnosis and benefits in image quality at the aortic root underscore the use of the tested high-resolution native 3D SSFP MRA as an appropriate alternative to contrast-enhanced MRA to assess the thoracic aorta.

## Funding

None.

**Figure 1:**
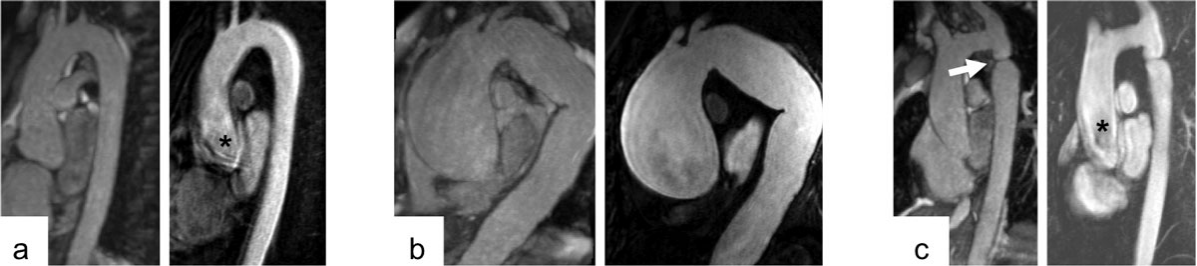
**Set of examples of the native MRA (left) and the contrast-enhanced MRA (right): a) Thoracic aorta with normal dimensions**. The sharpness of the aortic root achieved with the native MRA (left) is visible in comparison to the blurry borders provided by the contrast-enhanced MRA (*; right). b) Large ascending aortic aneurysm. c) Aortic coarctation (white arrow). Again the blurry aortic root with the contrast-enhanced MRA is recognizable (*; right).

